# Supplementary figures and images for: Dietary variations drive divergent phenotypic, transcriptomic, and metatranscriptomic profiles in Biomphalaria glabrata, a schistosomiasis vector snail
Source: Parasit Vectors. 2026 Apr 30;19:251. doi: 10.1186/s13071-026-07403-3 (PMC13276955; doi:10.1186/s13071-026-07403-3)

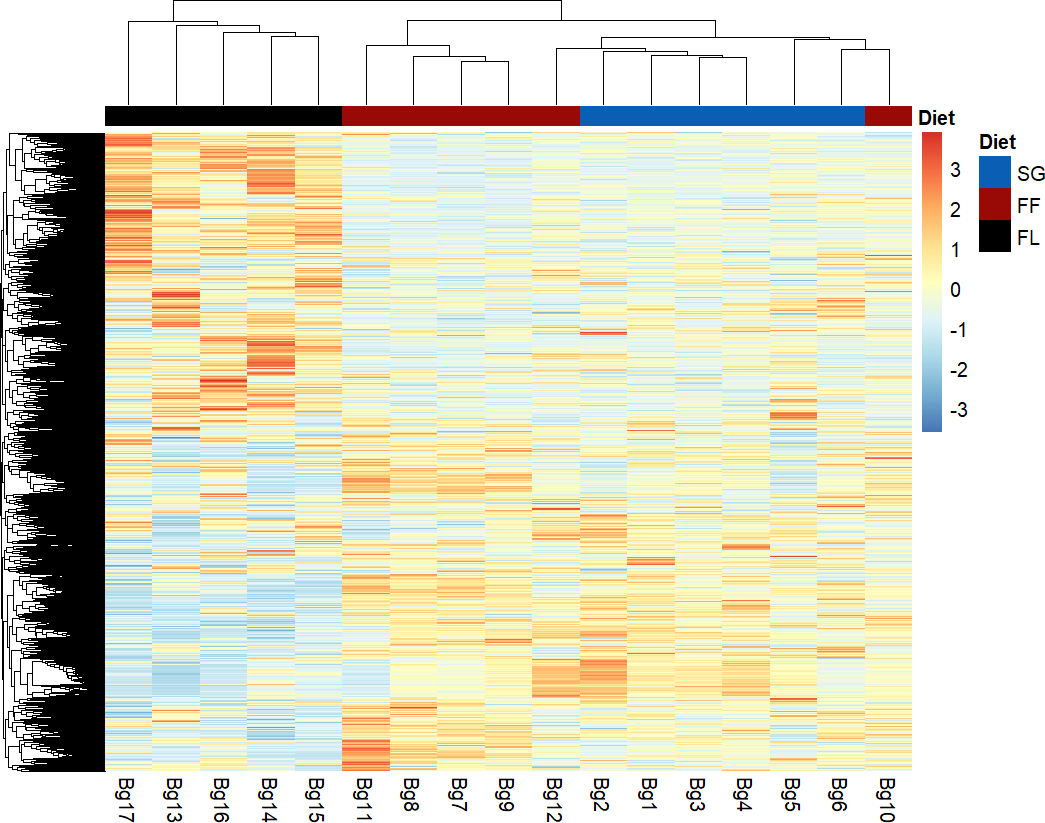

Supplement: Supplementary file 1 — Additional file 1. Fig. S1. Global heatmap for Biomphalaria glabrata gene expression across all samples based on dietary feeding. The colour gradient scale represents the Z-score (scaled) expression of a gene in a sample. Positive values indicate the gene expression in the sample is above the mean expression of the same gene across all samples. Negative Z-score indicates that the sample has lower expression for that gene compared with the gene’s average expression across all samples. At zero Z-score, expression of the gene in the sample equals the average expression of that gene across all samples. Therefore, the global heatmap only reflects relative gene expression patterns across conditions, not statistically significant up- or down-regulation between groups. [file 13071_2026_7403_MOESM1_ESM.tif]

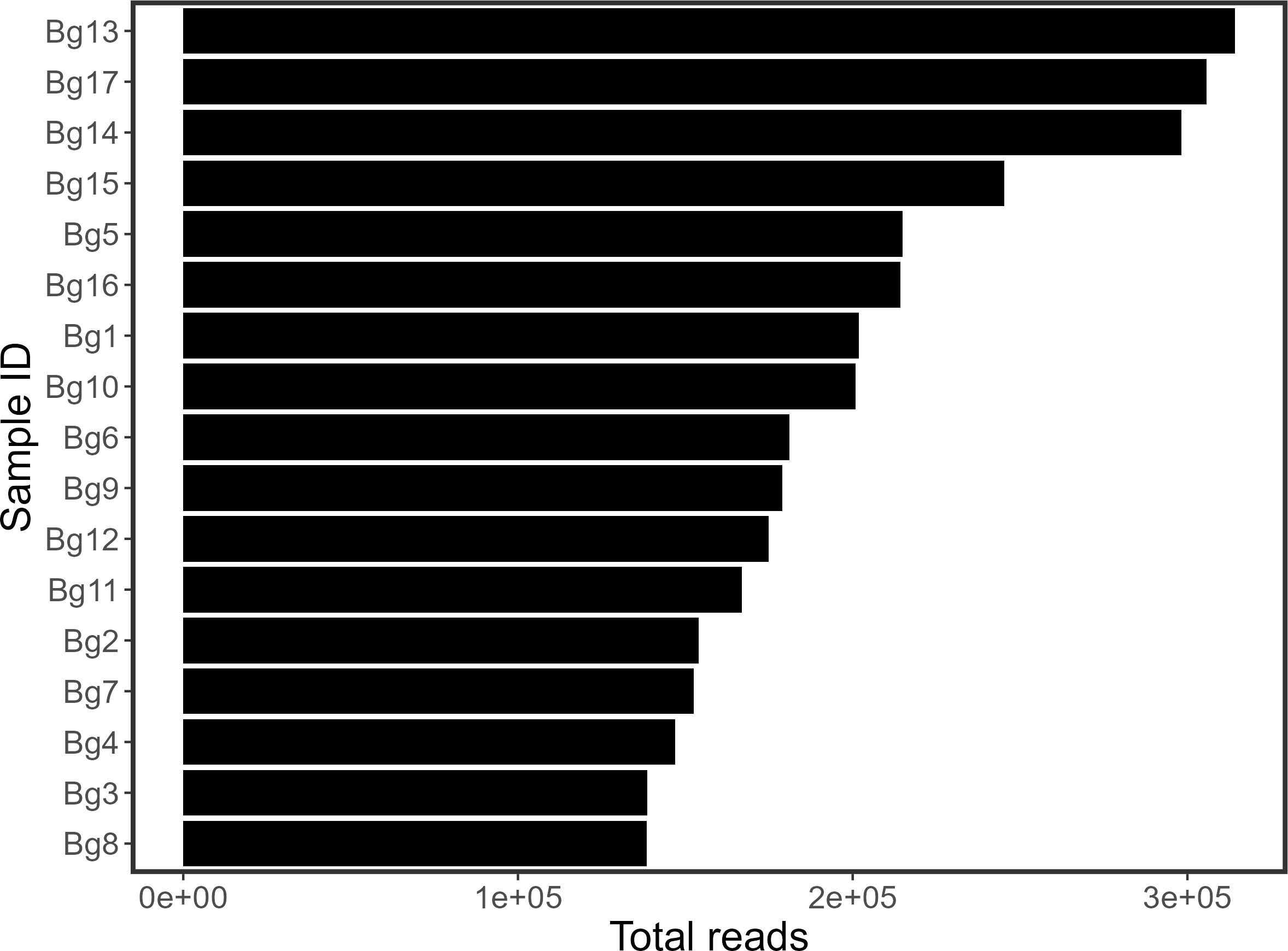

Supplement: Supplementary file 2 — Additional file 2. Fig. S2. Microbiome library size per sample. This was estimated based on raw operational taxonomic units (OTUs) and represents the total number of RNA sequence reads generated for a single biological sample. [file 13071_2026_7403_MOESM2_ESM.tif]
